# Influence of material property variability on the mechanical behaviour of carotid atherosclerotic plaques: A 3D fluid-structure interaction analysis

**DOI:** 10.1002/cnm.2722

**Published:** 2015-05-28

**Authors:** Jianmin Yuan, Zhongzhao Teng, Jiaxuan Feng, Yongxue Zhang, Adam J Brown, Jonathan H Gillard, Zaiping Jing, Qingsheng Lu

**Affiliations:** 1 Department of Radiology, University of CambridgeCambridge, CB2 0QQ, UK; 2 Department of Engineering, University of CambridgeCambridge, CB2 1PZ, UK; 3 Department of Vascular Surgery, Changhai HospitalChanghai Road, Shanghai, 200433, China; 4 Division of Cardiovascular Medicine, University of CambridgeCambridge, CB2 1TN, UK

**Keywords:** atherosclerosis, stroke, mechanics, stress, material property, sensitivity

## Abstract

Mechanical analysis has been shown to be complementary to luminal stenosis in assessing atherosclerotic plaque vulnerability. However, patient-specific material properties are not available and the effect of material properties variability has not been fully quantified. Media and fibrous cap (FC) strips from carotid endarterectomy samples were classified into hard, intermediate and soft according to their incremental Young's modulus. Lipid and intraplaque haemorrhage/thrombus strips were classified as hard and soft. Idealised geometry-based 3D fluid-structure interaction analyses were performed to assess the impact of material property variability in predicting maximum principal stress (Stress-P_1_) and stretch (Stretch-P_1_). When FC was thick (1000 or 600 µm), Stress-P_1_ at the shoulder was insensitive to changes in material stiffness, whereas Stress-P_1_ at mid FC changed significantly. When FC was thin (200 or 65 µm), high stress concentrations shifted from the shoulder region to mid FC, and Stress-P_1_ became increasingly sensitive to changes in material properties, in particular at mid FC. Regardless of FC thickness, Stretch-P_1_ at these locations was sensitive to changes in material properties. Variability in tissue material properties influences both the location and overall stress/stretch value. This variability needs to be accounted for when interpreting the results of mechanical modelling. © 2015 The Authors. *International Journal for Numerical Methods in Biomedical Engineering* published by John Wiley & Sons Ltd.

## 1. Introduction

Stroke is the leading cause of disability worldwide and remains a significant source of mortality [1]. Carotid atherosclerotic disease is responsible for around 30% of all ischemic strokes [2]. At present, carotid luminal stenosis is the only validated diagnostic criterion for patient risk stratification, but this measure becomes less reliable in patients with mild to moderate stenoses [3]. There is increasing evidence to suggest that the physical characteristics of atherosclerotic plaques, and the mechanical loading within the structure, may allow greater potential to discriminate clinical progression than luminal stenosis alone.

A vulnerable carotid atherosclerotic plaque is characterised by the presence of intraplaque haemorrhage (IPH) and a large lipid-rich necrotic core, with symptomatic plaques also showing evidence of fibrous cap (FC) rupture. These features can be accurately quantified by *in vivo* high-resolution, multi-contrast magnetic resonance imaging [4–6], which has been shown to predict future events in both symptomatic [7, 8] and asymptomatic, [9, 10] patients. IPH and FC rupture have also been identified as higher-risk features associated with both clinical symptoms at presentation [11]. As plaques are continually subject to mechanical loading, because of pulsatile blood pressure and flow, FC rupture is thought to occur when loading exceeds its material strength [12, 13]. FC stress can differentiate symptomatic from asymptomatic patients [14, 15] and has also been found to be associated with subsequent cerebrovascular ischaemic events in symptomatic patients [16].

There is therefore a need to integrate both plaque morphological/compositional features with the critical mechanical conditions, if patient risk stratification is to be improved. However, the reliability of computational modelling to calculate the critical mechanical conditions is dependent on the accuracy of the material properties for each atherosclerotic component, including intraplaque haemorrhage or thrombus (IPH/T), lipid and FC. Previous *ex vivo* studies have shown the wide variability of material properties from sample to sample [17–20], suggesting the need of patient-specific material properties. Although advanced imaging techniques may make this achievable [21], the accuracy of material properties derived from *in vivo* measures requires further comprehensive validation [22–24]. Additionally, a complex inverse procedure has to follow to estimate nonlinear mechanical properties of vascular tissues based on *in vivo* data [25, 26]. Because of these limitations, patient-specific material properties have not been considered in the majority of clinically oriented studies [13, 27–30]. Thus, there is a need to assess whether variability in tissue material property measures impacts the overall calculation of the critical mechanical conditions within an atherosclerotic plaque.

It has been previously shown that the variation in material properties generates relatively small errors in the prediction of structural stresses [31]. Even a ±50% variation in elastic modulus leads to <10% change in stress at the site of plaque rupture. Sensitivity to variations in the elastic modulus is comparable between isotropic nonlinear, isotropic nonlinear with residual strains and transversely isotropic linear models [31]. This was confirmed when the change in stress and strain values predicted, using parameters from two very different specimens, were relatively minor (within 20%) despite the substantial difference in stiffness and direction of anisotropy [32]. Conflicting results have suggested that peak stress varied largely with the intima stiffness, in particular, when the FC was thin [33]. This study aims to assess the influence of variability in material properties of media, FC, lipid and IPH/T obtained from direct material tests on stress and stretch conditions within the plaque structure.

## 2. Methods

### 2.1. Material test and tissue stiffness category

**Figure 1:**
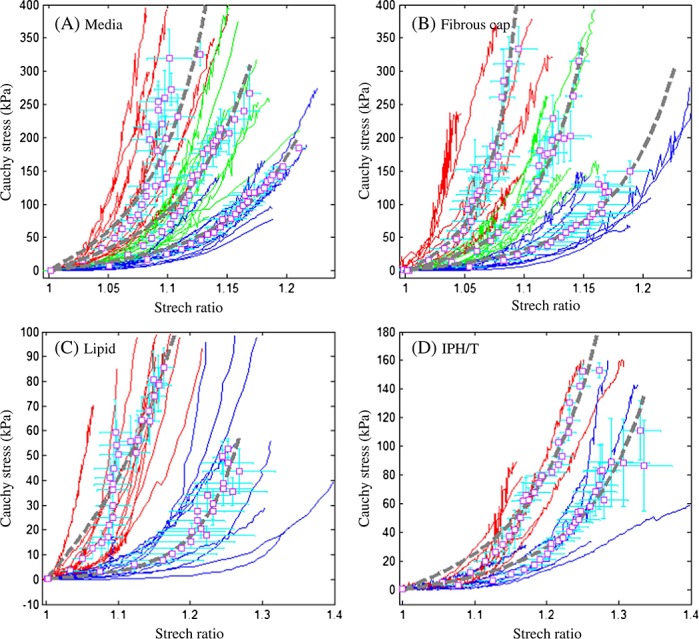
Stress–stretch curves obtained from uni-extension tests with different atherosclerotic components (A) media; (B) fibrous cap; (C) lipid and (D) intraplaque haemorrhage/thrombus. Curves in red stand for those classified as hard, green for intermediate and blue for soft. Points represent the energy-based averaged data for each stiffness category, and the associated dash line is the fitting line based on modified Mooney–Rivlin strain energy density function.

Carotid plaque samples were collected from 21 symptomatic patients who were scheduled from endarterectomy. Each sample was cut into rings along the axial direction and media; FC, lipid and IPH/T were further isolated from each ring, if present, to perform uni-extension testing. Full details regarding the testing procedure have been previously described [34]. In total, 65 media strips from 17 endarterectomy samples, 59 FC strips from 14 plaques, 38 lipid strips from 11 plaques and 21 IPH/T strips from 11 plaques underwent successful testing. The stretch–stress curves of each tissue type varied widely (Figure 1) with some of them being much stiffer than others. In this study, an incremental Young's modulus-based classification method was proposed to classify media and FC into three stiffness categories: hard, intermediate and soft; and lipid and IPH/T were classified as hard and soft considering the smaller number of tissue strips. The incremental Young's modulus, *E*, was calculated using


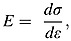


where *σ* and *ε* were Cauchy stress and strain described by modified Mooney–Rivlin strain energy density function,


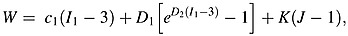


in which *I*_1_ and *J* are the first invariant and Jocabian determinate of the deformation gradient, respectively; *c*_1_ and *D*_1_ are material constants, and *K* is the Lagrange multiplier for incompressibility. The stiffness category was generated following steps of (1) the stress–stretch curve of each tissue strip was firstly fitted using the Mooney–Rivlin strain energy density function; (2) the incremental Young's modulus, 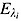 (*i* = 1,…100), was computed at 100 stretch levels that equally distributed between 1.0 and 1.4; (3) each 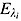 was scored by 1, 2 or 3 according to the ranking in media and FC and by 1 or 2 in lipid and IPH/T group; and (4) the score at each stretch level was finally summed up to represent the stiffness ranking of a tissue strip, and it was further classified as hard, intermediate or soft according to the total score. The upper 1/3 of media and FC were classified as hard followed by intermediate, while the lower 1/3 were classified as soft; for lipid and IPH/T, the upper 1/2 were classified as hard, while the remaining were classified as soft.

In each stiffness category, the total energy of each curve was computed by using [34]


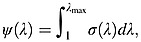


in which *λ*_max_ is the maximum stretch value of a stress–stretch curve. An energy-based average approach [34] was used to generate a representative curve for each stiffness catalogue. Fifty equal distance internals were placed between maximum and minimum energy levels, and the stretch and stress of experiment points within each energy level were averaged, as shown in Figure 1. The data points were fitted using modified Mooney–Rivlin strain energy density function to obtain the material constants for each stiffness category.

### 2.2. 3D fluid-structure interaction analysis

**Figure 2:**
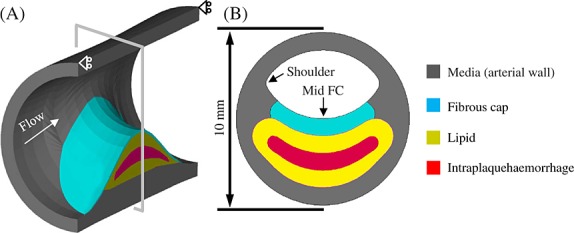
3D geometry of the idealised plaque model composed of fibrous cap, lipid and intraplaque haemorrhage (A) sagittal cut of 3D geometry and (B) the cross-section at the most stenotic site. The extended section is not shown, and fibrous cap thickness can be adjusted by moving the lipid and IPH towards and away from the luminal edge.

Three dimensional idealised plaque models were constructed to perform the parameter studies. As shown in Figure 2, the model represented a typical atherosclerotic plaque with 50% stenosis, composed of FC, lipid and IPH (the rest was assumed to be media). The plaque length was set to be 20 mm, and the lengths of proximal and distal sections were 120 mm to avoid potential entrance effect. As FC thickness <65 and <200 µm are the accepted definition of rupture-prone plaques in the coronary [35] and carotid [36] arteries, respectively, models with a cap thickness of 65 and 200 µm were constructed to represent high-risk lesions, and models with FC thickness 1000 and 600 µm were used to represent low-risk lesions.

**Figure 3:**
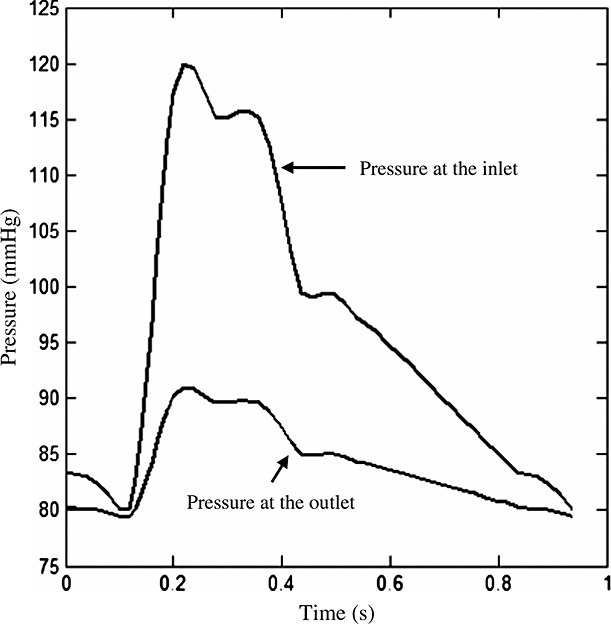
Inlet and outlet pressure waveforms.

Plaque components were assumed to be hard, intermediate and soft as described previously resulting in 36 plaque models with different combination of stiffness categories for each case with a defined FC thickness. The total number of models was therefore 144 (=36 × 4). A ‘volume curve fitting’ technique [13] was employed to assist the generation of hexahedron elements for both structure and fluid domains, to handle fluid-structure interaction (FSI) analyses involving non-linear material properties and big deformations. The pressure waveforms at the inlet and outlet are shown in Figure 3, with systolic and diastolic pressures at the inlet being 120 and 80 mmHg, respectively. Symmetric conditions were adopted as shown in Figure 2A. The blood flow was assumed to be Newtonian, viscous and incompressible. FSI simulations were performed using ADINA 8.7 (ADINA Inc., MA, USA). The energy convergence criterion was used for solid domain during equilibrium iterations with the relative energy tolerance being 0.05 and relative force and moment tolerance being 0.01. For the fluid domain, the relative tolerances for velocities, pressure and displacements were set to be 0.06 for controlling the equilibrium. The fluid-structure coupling was solved iteratively. Both the displacement and velocities at the fluid-structure interface and the forces on the structure due to the viscous fluid were checked for convergence. Relative displacement/velocity and force tolerances were both set to be 0.06. Maximum principal stress (Stress-P_1_) and stretch (Stretch-P_1_), both at mid FC and the shoulder region, were used to characterise the critical mechanical conditions within the plaque structure. The influence of material properties from different stiffness categories on both of Stress-P_1_ and Stretch-P_1_ was subsequently analysed.

## 3. Results

### 3.1. Material constants of different stiffness category

As shown in Figure 1, the modified Mooney–Rivlin strain energy density function could characterise the nonlinear stress–stretch curves for each tissue type. Moreover, the incremental Young's modulus-based scoring method could reasonably classify plaque component into different stiffness categories. The corresponding fitting parameters are listed in Table I.

**Table I:** Material constants of different stiffness categories for each tissue type.

Tissue type	Stiffness category	*c*_1_ (×10^−3^ kPa)	*D*_1_ (kPa)	*D*_2_
Media	Soft	69.37	3.59	11.51
	Intermediate	335.90	6.95	14.32
	Hard	0.01	7.58	22.43
Fibrous cap	Soft	159.86	2.62	13.57
	Intermediate	108.41	3.11	25.54
	Hard	31.71	2.43	62.13
Lipid	Soft	129.64	0.34	11.71
	Hard	124.90	11.13	5.28
Intra-plaque haemorrhage or thrombus	Soft	148.21	2.42	5.60
	Hard	147.70	6.10	5.88

### 3.2. Material properties uncertainty analysis

**Figure 4:**
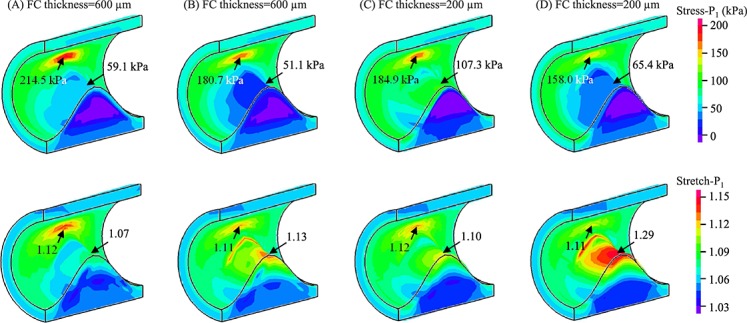
Band plots of Stress-P_1_ and Stretch-P_1_ for idealised models with different combination of stiffness categories and fibrous cap (FC) thickness (A) fibrous cap thickness – 600 µm, media and FC – intermediate, lipid and intraplaque haemorrhage – soft; (B) the same model as the one shown in A, but fibrous cap – soft; (C) the same model as the one shown in A, but fibrous cap thickness – 200 µm; and (D) the same model as the one shown in B, but fibrous cap thickness – 200 µm.

Figure 4A shows band plots of Stress-P_1_ and Stretch-P_1_ in a typical plaque structure with intermediate media and FC, and soft lipid and IPH/T (FC thickness = 600 µm). High stress and stretch concentration were found in the shoulder region where luminal curvature was large. If the FC was changed to be soft (Figure 4B), both stress and stretch in the shoulder region decreased by 15.8% and 0.9%, respectively, and the values at the mid FC decreased by 13.5% and increased by 5.6%. Alternatively, if the material properties were kept unchanged, but FC thickness reduced to be 200 µm, the stress at the mid FC increased by 81.6% (Figure 4C). As shown in Figure 4D, when a soft FC thickness was reduced to 200 µm, the stretch at the mid FC increased by 20.6%.

**Figure 5:**
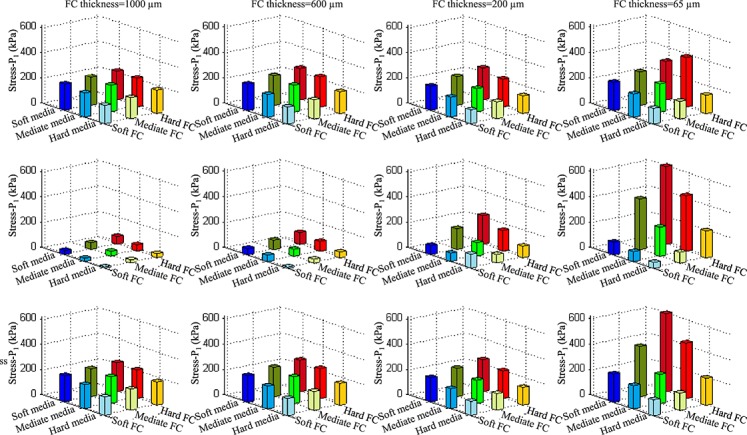
Stress-P_1_ in the shoulder and at mid fibrous cap (FC) with different combinations of media and FC stiffness categories under the condition of both lipid and intraplaque haemorrhage being soft (first row: Stress-P_1_ in the shoulder region; second row: Stress-P_1_ at mid FC; third row: critical stress, maximum value of Stress-P_1_ in the shoulder and at mid FC, and FC thickness for the first, second, third and fourth columns are 1000, 600, 200 and 65 µm, respectively.

It can be seen from Figure 4 that the material properties produce different effects on the stress and stretch at each location (the shoulder region and mid FC). Such influence depends largely on FC thickness with more details shown in both Figures 4 and 5. When the FC was thick (1000 or 600 µm; the first two columns in Figures 4 and 5), high stress and stretch concentrations were mainly located at the plaque shoulder. For FC 1000 µm, the highest stress and stretch concentrations (228.5 kPa and 1.240) were found through the combination of soft media and hard FC (both lipid and IPH were soft); and the lowest stress and stretch concentrations (146.5 kPa and 1.06) were for hard media and all other components being soft. Similar stress and stretch characteristics were found in the case of FC 600 µm. The highest stress and stretch concentrations were 249.8 kPa and 1.225, respectively, under the combination of soft media and hard FC (both lipid and PH were soft), and the lowest of 136.0 kPa and 1.052, respectively, under the combination of hard media and all other components being soft. Big stress and stretch changes were found at mid FC when different combinations of material strength were applied. When FC was 1000 µm, the stress at the mid FC could change from 9.3 kPa in the case of media being hard while all others soft to 62.9 kPa in the case of media, lipid and IPH being soft and FC hard. When was 600 µm, the stress at the mid FC could change from 10.7 to 94.0 kPa using the exact same stiffness categories. When the FC thickness reduced to 200 µm, the stress level at the mid of FC increased, while if FC thickness reduced to 65 µm, the highest stress concentration shifted from the shoulder to mid FC, in most combinations. As shown in the chart at the middle of the last column in Figure 5, stress could rise to 615.2 kPa when the media was soft and FC hard (both lipid and PH were soft).

**Figure 6:**
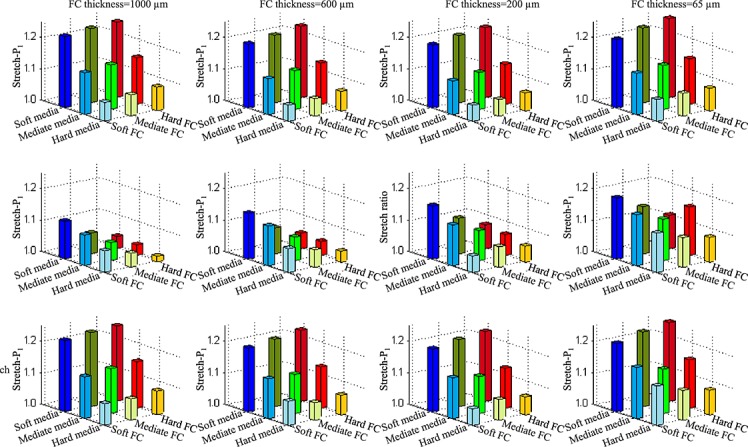
Stretch-P_1_ in the shoulder and at mid FC with different combinations of media and FC stiffness category under the condition of both lipid and intraplaque haemorrhage being soft (first row: Stretch-P_1_ in the shoulder region; second row: Stretch-P_1_ at mid FC; third row: critical stretch, the maximum value of Stretch-P_1_ in the shoulder and at mid FC, and FC thickness for the first, second, third and fourth columns are 1000, 600, 200 and 65 µm, respectively.

It can been seen from the last row of Figure 5, the impact of combinations of different material stiffness's on critical stress concentrations, defined as the maximum value of stress in the shoulder and at mid FC, becomes greater when FC thickness reduces. The stress standard deviation increased from 28.4 kPa for FC 1000 µm and 40.2 kPa for FC 600 µm to 47.5 kPa for FC 200 µm and 163.8 kPa for FC 65 µm. The critical stretch concentrations are also shown to be sensitive in all cases, either with a thick or thin FC (the last row in Figure 6). The stiffness of media dominates stretch conditions and the largest critical stretch, defined as the maximum stretch value in the shoulder and at mid FC, always appeared in cases with a soft media. Under most conditions, large stretch concentrations were located in the shoulder region (the first row in Figure 6) with some exceptions (shift to the mid of FC) when FC thickness reduced to be 200 or 65 µm.

**Figure 7:**
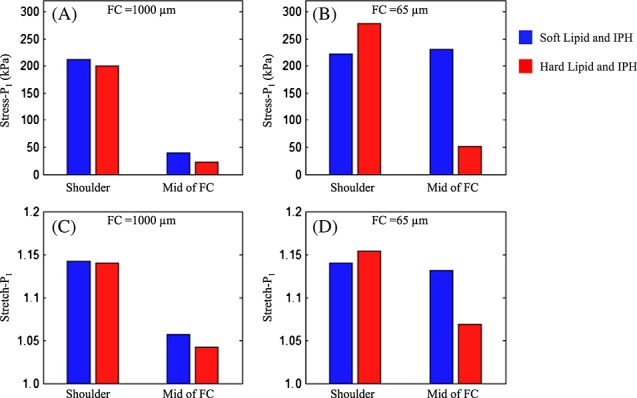
Comparisons of Stress-P_1_ and Stretch-P_1_ when lipid and intraplaque haemorrhage (IPH) are soft and hard (A) and (B) Stress-P_1_ in the shoulder and at mid fibrous cap (FC) when FC thickness is 1000 and 65 µm, respectively; and Stretch-P_1_ in the shoulder and at mid FC when FC thickness is 1000 and 65 µm, respectively.

Apart from media and FC, the stiffness of lipid and IPH play an important role on the pattern of stress and stretch distribution within the structure. When FC was thick, for a fixed combination of media and FC, stress and stretch in the shoulder region and at mid FC were insensitive to the stiffness of lipid and IPH. However, when FC thickness decreased, stress and stretch at mid FC increased dramatically. As shown in Figure 7A and B, for the combination of intermediate media and FC, when FC was 1000 µm, stress increased from 201.4 to 211.8 kPa in the shoulder and 23.2 to 40.4 kPa at mid FC when the hard lipid and IPH were replaced by soft. Under the same circumstance, when FC 65 µm, stress decreased from 278.4 to 222.3 kPa at the shoulder and increased by 353% at mid FC (from 51.0 to 231.0 kPa). Similar observations were found in terms of stretch as shown in Figure 7C and D.

## 4. Discussion

Our study aimed to explore the influence of material property variability on critical plaque stress and stretch conditions, by using different combinations of stiffness categories obtained from direct uni-extension material tests. The results obtained show that the sensitivity of stress to tissue material properties largely depends on FC thickness and location. Generally, in the shoulder region (first row of Figure 5 and 6), softer media results in higher stress and stretch; and harder FC leads to higher stress and stretch. Compared with the plaque shoulder, stress at mid FC was more sensitive to changes in material properties even when the FC was thick (the second row in Figure 5). Except for some combinations when FC 200 µm (the third chart in the second row), although the percentage change was large, changes in absolute stress values were relatively small (<80 kPa). In the case of thin FC, the change of stress both in the shoulder and at mid FC was large when different combinations of stiffness categories were applied. In particular in the case of FC thickness being 65 µm, at mid FC, the average stress value was 245.8 kPa with standard deviation of 197.1 kPa (the last chart in the second row of Figure 5). The stretch either in the shoulder or at the mid of FC appeared to be always sensitive to alterations in plaque material properties.

In general, stress and stretch within FC were sensitive to the uncertainty of material stiffness, which is in agreement with a reported study by Akyildiz *et al* [33]. However Williamson *et al* [31] concluded that the variation in material properties generated relatively small errors in the prediction of structural stresses. These contradictory views may be because of the relatively smaller variation of material stiffness used [31], compared with the current study. Moreover, the material stiffness of lipid used in the study performed by Williamson *et al* was significantly underestimated as compared with the value obtained from direct measurements [34], being derived from an experimental study using polymers mimicking lipid pools [37]. In the case of stretch ratio being 1.25, the stress was only 1.98 kPa, while it should be about 100 kPa according to the direct measurement using lipid tissue strips [34]. When the lipid was changed from hard to soft, the change of stress was 3.79 kPa at the stretch level of 1.25. Such small changes were unlikely to have a significant impact on the stress level within FC.

In this study, FC thicknesses were chosen to be 1000, 600, 200 and 65 µm as each of these values represented a predefined threshold. In our study, 1000 µm was used to represent lesions with a thick FC, while 600 µm was the mean minimum FC thickness quantified by *in vivo* high-resolution magnetic resonance imaging in symptomatic patient groups [6]. Based on autopsy specimens, 200 µm was suggested as the optimum value for FC thickness to allow discrimination between ruptured and non-ruptured carotid plaques [36], while 95% of all ruptured coronary plaques have been found to have a cap thickness of <65 µm [35]. In this study, the idealised plaque model was reconstructed referring to the dimension of human carotid plaques. The level of stress concentration obtained was comparable with previous reported patient-specific analyses of lesions from the same location. For example, Tang *et al* [13] analysed stress conditions within five ruptured human carotid plaques. He and his colleagues assumed that FC thickness at the site of rupture was 200 µm. Stresses at the ruptured site were 457.85, 241.90, 195.51, 161.92 and 179.14, respectively (Mean ± SD: 247.3 ± 121.4 kPa). These values were comparable with the case of FC thickness = 200 µm in our study (the third column in Figure 5).

As shown in Figures 4 and 5, we observed higher stress and stretch levels in models with a soft media. It has been demonstrated that arterial stiffness increases with age [38], implying that lesions in younger populations may be subject to higher mechanical loading. However, this does not necessarily mean that atherosclerotic lesions in younger populations are at a higher risk of rupture, as other pathobiological factors, including local inflammation [39], are important in plaque instability. Moreover, we found that stress was increased when FC became harder. In the shoulder region, this may be because of the mismatch of material properties as this is known to result in high stress concentrations [29], while in mid FC this may be because of stronger material undertaking bigger loading. Variations of stress and stretch, with changing of different combination of stiffness categories, may represent some underlying pathological process. Indeed, it has been observed that hypocellular FCs were one to two times stiffer than cellular caps, with calcified caps being four to five times stiffer [40]. As has been shown in this study, stiffer FC could act to increase stress and stretch, which may explain why lesions with a hypocellular FC are at the highest risk of rupture. Moreover, clinical trials have shown that lipid-lowering therapy may reduce the incidence of cardiovascular events [41, 42] by transforming cholesterol esters to cholesterol monohydrate. This would lead to a stiffer lipid core [43] and decrease the stress and stretch level within the cap, in particular when FC is thin (Figure 7). Furthermore, the results shown in Figure 7 are consistent with previous findings that when fresh haemorrhage progressed to be chronic (stiffer), FC stress decreased by 35% [44].

Because of the difficulty in obtaining patient-specific material properties, a single set of material constants for each plaque component has been used in most reported computational modelling studies. However, experiments have shown that the material properties of plaque components are not only patient dependent but also location dependent [45–49, 43, 34]. In addition, this study suggests that the variation of material properties may have an appreciable influence on simulation results, and errant material parameters have potential to lead to inaccurate mechanical analyses. If mechanical modelling is to be improved, there is a need to obtain patient-specific material properties by using *in vivo* imaging modalities [21]. However, comprehensive understanding on the material behaviour of each atherosclerotic component from *in vivo* measurements requires validation and remains constrained by current imaging limitations such as poor resolution, scanning time and signal penetration depth. In the meantime, this study might provide a compromised solution: (1) plaque stiffness could be classified as hard, intermediate or soft according to *in vivo* measurements, such as elastography and (2) the material constants suggested for each stiffness category, as listed in Table I, could be used to perform mechanical analysis. However, it is challenging to estimate the stiffness of each component based on elastography alone. Novel MR deformation imaging techniques [50, 51] providing the combination of elastography map and anatomic information may be a future potential solution.

## 5. Limitation

Within this study, only Stress-P_1_ and Stretch-P_1_ were analysed as these stresses are believed to play a role in plaque rupture, compared with blood shear stress [13]. In cases of patient-specific analysis, the 3D structure-only model rather than 3D FSI can be used to re-predict these two parameters as it is inexpensive yet reasonably accurate approximation [52]. The conclusions were obtained based on idealised plaque geometries for the convenience of parameter studies. Further studies are required to validate these conclusions using patient-specific plaque geometries. We made no attempt to measure calcification during uni-axial testing or included this component in modelling processes. However, arterial calcification is considering a protective mechanism for rupture and ruptured plaques are unlikely to contain large plates of calcium [53], although plaques with juxtaluminal calcific nodules may be at higher risk [29]. We acknowledge other mechanisms may be involved in ischemic events, including calcific nodules and erosions. The effects of material properties on these mechanisms are yet to be determined. The material properties of media, FC, lipid and IPH/T were obtained from uni-extension tests, and anisotropic material behaviour was not considered. Finally, no attempt was made to model residual stresses.

## 6. Conclusions

The sensitivity of stress at either the shoulder region or mid FC to tissue material properties largely depends on FC thickness. In the shoulder region, softer media results in higher stress and stretch; and harder FC leads to higher stress and stretch. Compared with the plaque shoulder, stress at mid FC is more sensitive to changes in material properties even when FC is thick. The stretch either in the shoulder or at the mid of FC appears to be always sensitive to material properties.
